# Adolescent Sexual and Reproductive Health During the COVID-19 Pandemic: A Mini Review

**DOI:** 10.3389/frph.2022.794477

**Published:** 2022-03-30

**Authors:** Candice Groenewald, Nazeema Isaacs, Dane Isaacs

**Affiliations:** ^1^Centre for Community-Based Research, Human Sciences Research Council, Durban, South Africa; ^2^Psychology Department, Rhodes University, Grahamstown, South Africa; ^3^Human and Social Capabilities, Human Sciences Research Council, Cape Town, South Africa

**Keywords:** sexual and reproductive health (SRH), adolescents, teenage pregnancies, SRH services, sexual violence, contraceptives

## Abstract

This mini review explores the impact of the COVID-19 pandemic on adolescent sexual and reproductive health. We conducted a rapid review of the literature across three databases, with a particular focus on the African continent. Few studies have specifically focused on adolescents in Africa and this paper contributes to this paucity of research. Findings revealed the unintended consequences of the pandemic. Studies across several countries showed that the respective lockdown measures restricted adolescents' access to sexual and reproductive health services. The literature also showed increases in adolescent pregnancies during the lockdown, along with increases in reports of sexual violence against adolescents. We conclude this paper by offering recommendations to address these unintended consequences and potentially improve adolescent sexual and reproductive health in African communities.

## Introduction

Sexual and reproductive health (SRH) is a human rights issue that is important for human and sustainable development because of its association with gender equality and the health and wellbeing of newborn babies, children, adolescents, and women (1, 2). The 2030 Agenda for Sustainable Development and the movement toward universal health coverage have been essential in the promotion of Sexual Reproductive Health Rights (SRHR) with a focus on specific aspects of SRHR, including contraception, maternal and newborn health, and HIV/AIDS. Over the past few decades, countries around the world have made remarkable gains in the promotion of SRHR and improving access to SRH services. However, the gains have been inequitable, particularly within developing countries where services have often fallen short in coverage and quality. In African countries, for example, gender inequality, poor infrastructure, poverty among women, limited economic resources and increased levels of violence against women, particularly the genital mutilation of women, have significantly impeded on the access of women and adolescents to SRH services (3). Adolescents in Sub-Saharan Africa (SSA) encounter of the most significant SRH challenges and endure the highest burden of adverse SRH outcomes (4). For instance, majority of new HIV-infections in East and SSA, are found among adolescent girls (4). Globally, young women make up more than 60% of all young people living with HIV, and in Sub-Saharan Africa that rate increases to 72% (5). Additionally, child marriages are on the increase in West Africa, and adolescent girls in SSA have limited access to modern contraceptives (4), contributing to increases in adolescent pregnancies. The prevalence of adolescent pregnancies in Africa before the COVID-19 pandemic has been estimated at 18.8% (6).

Nevertheless, the last decade has seen increased interest and awareness of the sexual and reproductive health of women and adolescents in Africa (7, 8). Many have defined this as a state of wellbeing where adolescents have rights to be free from sexual violence, unwanted pregnancies, unsafe abortions, have access to “the highest attainable standard of health” (standard 27), are protected from sexually transmitted infections, and have access to information and education related to sexual and reproductive health (9). In this regard, adolescent SRH entails access to services, support and education that advance their safety and freedom of choice. There has been progress made in SSA with regards to developing reproductive health policies and reforming laws to provide a framework for the implementation of SRHR programmes (10). For instance, the Maputo Plan of Action (MPoA) was implemented between 2008–2009. An evaluation of the implementation of the Maputo Plan of Action found that although most countries have formulated SRH policies, they have not necessarily translated these into the provision of services. In response to the limitations of MPoA, a Revised Maputo Plan of Action (MPoA) 2016–2030 was introduced at the 27th AU Summit in July 2016. The Revised MPoA aims to reinforce the call for universal access to comprehensive sexual and reproductive health services in Africa and lays foundation to the Sustainable Development Goals (10).

The COVID-19 pandemic, however, has significantly impacted the health and wellbeing of the global population. In addition to compromised physical health, the impact of the pandemic (and lockdown regulations) has been multi-systemic, leading to diminished societal mental health, restricted access to healthcare and support services, and increased economic precarity (11–13). Vulnerable populations like adolescents have been particularly affected by the lockdown, which has limited their access to education, psycho-social support services and placed many at risk of domestic violence (14). Experts have also called attention to the detrimental impact the pandemic may have on the SRH of adolescents, projecting increases in teenage pregnancies alongside decreases in access to SRH services, including safe abortion, and contraceptives, particularly access to condoms (15–17).

Against this backdrop and recognizing the negative impacts that previous epidemics have had on adolescent SRH (15, 18–20), we attempted to synthesis the literature that describe the impact of the current pandemic on adolescent SRH in Africa. In this paper, we conducted a mini review of the literature to understand the impact of the COVID-19 pandemic on SRH of adolescents in Africa.

### Review Approach

A systematic search was conducted across three database, namely EBSCO-host web, SAGE journals and Google Scholar using the search terms “sexual reproductive health,” (AND), “adolescents OR teenagers OR teen OR youth,” (AND) “COVID-19 OR coronavirus OR pandemic OR lockdown,” (AND) Africa. The time parameters of the search were January 2020 to August 2021. EBSCO-host web, like Google Scholar, is an aggregator database, meaning it includes content from various publishers (i.e., different journals and databases). These two databases were thus preferred in this rapid review as they provided access to a large scope of work, which might have been excluded in more focused databases. SAGE journals were included as it is a database that covers social and behavioral sciences research, including our interest in adolescent and youth studies through, for example, the Journal of Adolescent Research, Journal of Early Adolescence, Youth and Society and South African Journal of Psychology.

**Figure 1:**
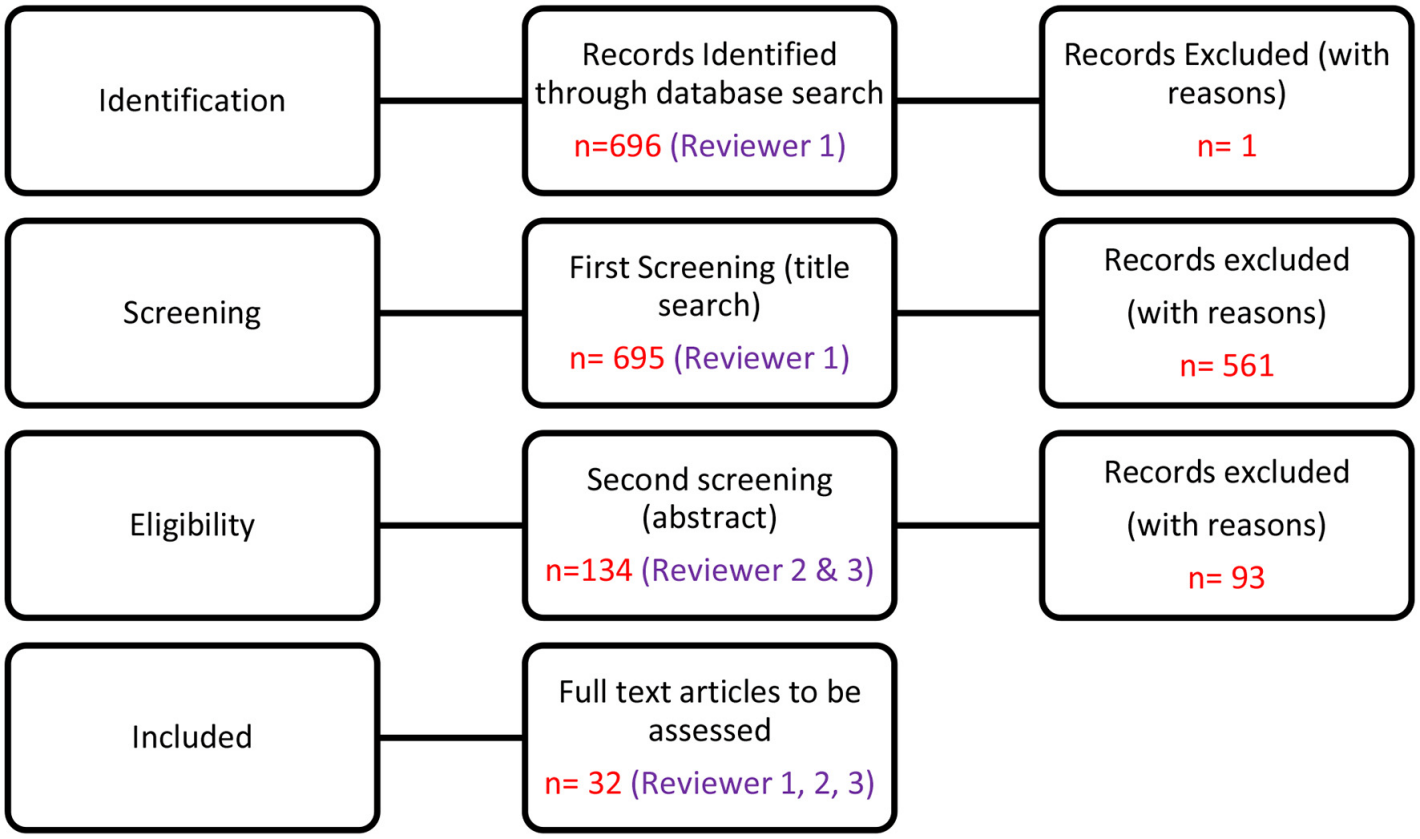
PRISMA diagram to screen articles.

EBSCO-host web produced 156 articles, SAGE Journals produced 1 and Google Scholar produced 538 articles. Articles were screened and only published articles (and reports) that had these keywords in the title, keywords or abstract (where relevant) were considered in the current paper. Articles selected for retrieval were independently assessed by two authors for methodological rigor. Two reviewers were included to avoid bias. Both reviewers consulted the necessary literature to familiarize themselves with the process. Working together in pairs enabled verification and contributed to the highest possible level of methodological rigor for this paper (see Figure 1).

**Figure 2:**
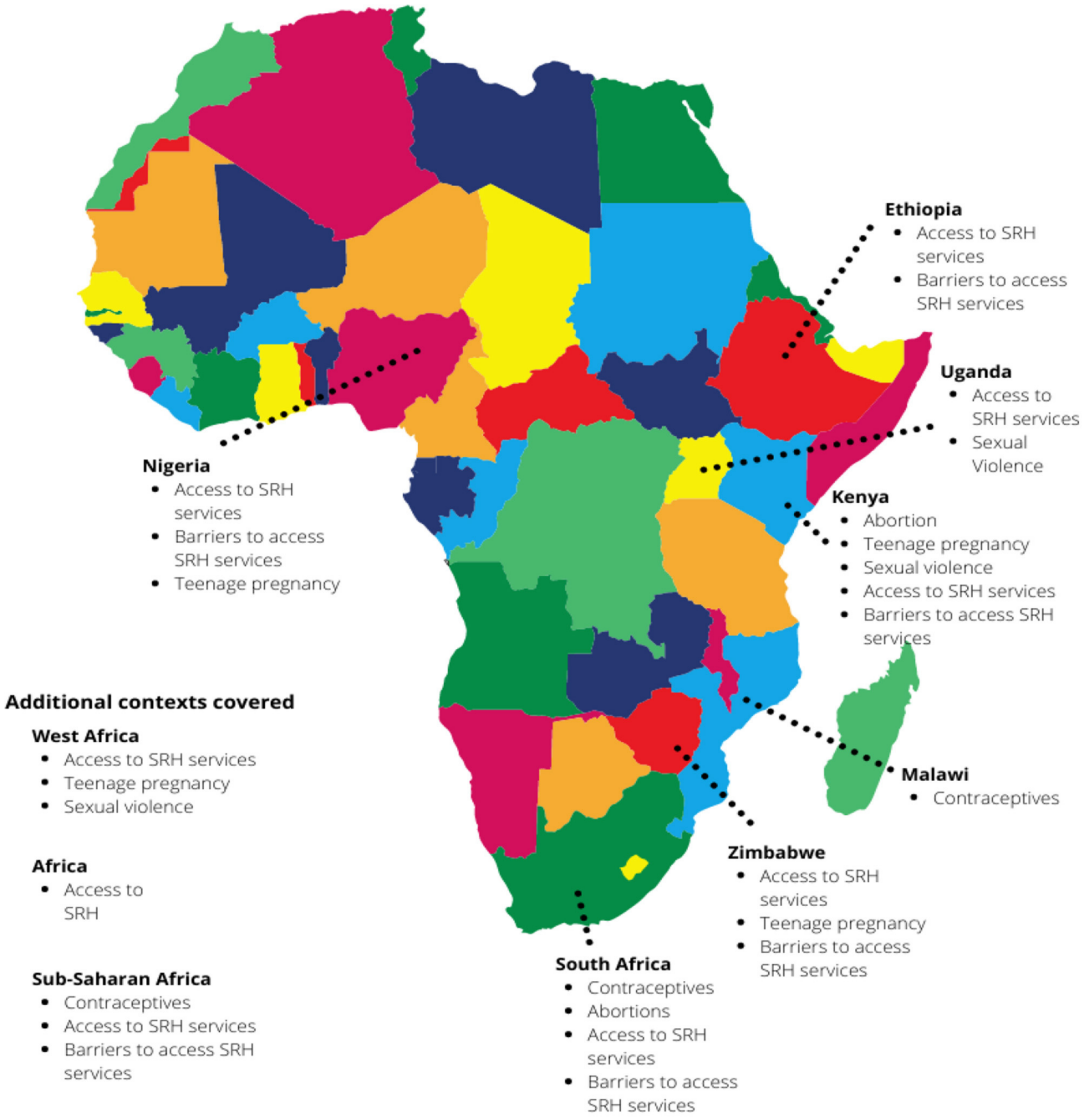
Map of countries included in this mini review.

The review included 32 articles (see Appendix A). As shown in the figure below, the articles spanned 8 African countries, while a few papers covered multiple contexts (see Figure 2). Majority of the papers were empirical (*n* = 19), followed by commentaries (*n* = 5), literature reviews (*n* = 4), letters to editors (*n* = 2) and policy briefs (*n* = 2). The results in the current paper will focus only on empirical papers (*n* = 19).

## Results

The primary foci of the studies included in this paper were directed toward teenage pregnancy, sexual violence, abortion, and the barriers toward accessing SRH services during the pandemic (including contraceptives). We conclude this paper by describing the strategies that researchers have prioritized to mitigate the impact of the pandemic on the SRH of adolescents in African communities.

### Increases in Teenage Pregnancy

The literature raised concerns over the increases in teenage pregnancies that were observed during the pandemic (21–23). In Kenya, most of the schools had more than 11 preexisting teenage mothers and the number of teenage mothers were expected to increase during the COVID-19 pandemic (22). Qualitative research with social workers in Zimbabwe also express concerns for the SRH of adolescents, highlighting issues of teenage pregnancies and increases in child marriage in response to the economic strain placed on families (23).

### Sexual Violence

The literature described increases in child sexual abuse in African countries during the pandemic. In Kenya, for example, research shows that the economic impacts of the pandemic and social restrictions of the lockdown leaves women and girls vulnerable to domestic violence, increased risk of sexual exploitation, and may increase child marriage amongst girls (22). Similar concerns were raised in Uganda where sexual violence was the “third most reported form of child abuse contributing 20.1% of all the cases” (24). Cases of sexual violations amongst girls during the 2020 lockdown have also been found in West Africa, including Benin, Cameroon, Côte d'Ivoire, Gambia, Ghana, Guinea, and Sierra Leone (25).

### Unsafe Abortions

Unsafe abortions are a particular high risk during the pandemic (26). Adelekan et al. (27) conducted a study in South Africa, and their findings revealed that termination of pregnancies (TOP) services had started to decline before and during the national lockdown due to COVID-19. Based on research conducted by Rohwerder (26), several girls in low- and middle-income countries indicated how access sexual and reproductive services were diverted. In this case, a 10% service disruption was projected to lead to 3.3 million unsafe abortions and an additional 1,000 maternal deaths (26).

### Barriers to Access SRH Services During the COVID-19 Pandemic

Majority of the articles in our dataset also described the barriers to accessing SRH services for adolescents during the pandemic. The COVID-19 pandemic and related lockdown significantly restricted access to adolescent friendly healthcare, labor clinics, and emergency services (21–23, 28). Research in Ethiopia found that, due to restricted access to healthcare facilities, home births were commonly reported during the pandemic (28). Similarly, research in Kenya also showed significant decrease in early access of antenatal care. With this said, however, there was a significant increase in women accessing skilled delivery in Kenya (29). In a few articles in our database, access to contraceptives was also discussed. In their report, Rafaeli and Hutchinson (21) revealed that girls from low-income countries experienced limited access to modern contraceptives as stocks ran significantly low during the COVID-19 pandemic. This was also the case in South Africa and Nigeria. In terms of South Africa, two studies by Bolarinwa (30) and Adelekan et al. (31) found that the COVID-19 pandemic had impeded on South Africans access to contraceptives and contraceptive technologies. Bolarinwa (30) found that two in every ten South Africans experienced limited access to condoms during the COVID-19 pandemic. In stark contrast to South Africa, Sitalire (32) and Karijo (33) found that the COVID-19 pandemic did not significantly impact on access to contraceptives in Ethiopia and Kenya.

In addition to contraceptives, articles in our sample also examined African adolescent girls' access to menstrual hygiene products during the pandemic. In Kenya, for instance, Masago and colleagues examined the impact of the pandemic on the welfare of Maasai girls (22). Their research showed that the Maasai girl child faced many SRH vulnerabilities such as bad menstrual hygiene, female genital mutilation, domestic violence, teenage pregnancy, and early marriages (22). As a result of the economic impact of the pandemic, Masago et al. (22) found that some Maasai girls engaged in transactional sex with men in exchange for sanitary towels as their guardians were unable to purchase these necessities. Similar findings were also shown in Zimbabwe, where Ndhlovu and Thembo (23) put forth that the national lockdown and restrictions had increased sexual reproductive health risks for women and girls in rural Zimbabwe. For example, during the national lockdown some hospitals and clinics in Zimbabwe provided limited access to non-COVID-19 patients. This acted as a barrier for women and girls who desired to access menstrual hygiene products and SRH services. The limited access to menstrual hygiene products was argued to result in increased health risks, such as unplanned and unwanted pregnancies and birth complications (23).

## Discussion

The aim of this mini review was to explore the impact of the COVID-19 pandemic on adolescents SRH in the African context. Our findings revealed that adolescents', and particularly females, lives were significantly impacted by the pandemic, and resonate with previous studies which show that adolescents' SRH have been compromised by the pandemic (12, 14–16).

The studies in our sample raised concerns over the increases in teenage pregnancies that were observed during the pandemic (21–23). This coincides with existing literature reports, where Kenya for example, noted a significant increase of more than 80% in teenage pregnancies in 2020 compared to 2019 (34). Similarly, in Malawi, in 2020, an approximately 35% rise was noted in the number of teenage pregnancies among adolescent girls (35). Furthermore, in Uganda, the amount of pregnant adolescent girls' identified doubled in 2020 (36).

Increases in child marriages, which is subsequently linked to teenage pregnancies, were also reported in the studies in this mini review. Previous literature shows that in four Eastern and Southern African countries (Ethiopia, Mozambique, Uganda, and Zambia), adolescent girls were severely impacted by the pandemic (37). In this regard, an increase in child marriage and teenage pregnancies, largely due to school closures and limited access to sexual and reproductive health services, were reported (37). Others have also prioritized the needs of girls in sub-Saharan Africa, where it is estimated that teenage pregnancies will observe a 65% increase due to the pandemic and that about one million girls will drop out of school (38, 39). Teenage pregnancies are associated with a higher likelihood of death because of childbirth complications and thus interventions and protection for vulnerable girls during the pandemic and beyond is imperative (37).

Linked to risks of teenage pregnancies, studies also highlighted the potential increase in unsafe abortions by adolescent girls during the pandemic (40). Considering that access to SRH services were limited during the pandemic, the impact of this restricted access was also flagged in other studies as having detrimental consequences for females who require abortion services. Feyissa, Toly and Ezeh (41) commented that reduction of SRH services in Ethiopia alone may result to more than three million unsafe abortions and one thousand maternal deaths due to unsafe abortions. These unintended consequences have also been highlighted across the globe where access to safe abortion facilities have been identified as a key intervention and policy priority worldwide (42–46).

Furthermore, the studies in this mini review also reported increased levels of sexual violence perpetrated against adolescent girls during the COVID-19 pandemic. The literature showed that economic pressure and social restrictions enhanced adolescent girls' vulnerabilities to domestic violence during lockdown periods in Africa. Similar findings have been reported during previous epidemics. For example, during the 2014–2016 Ebola outbreak, economic vulnerability, and school closures made girls and women vulnerable to increased acts of sexual violence, coercion, and exploitation (47, 48). Relatedly, reports have also shown how gender-based violence (GBV) perpetrated against girls and women in Africa, have intensified during the Covid-19 pandemic (49). A recent report published by the African Union Commission in collaboration with other organizations (2020) indicated a sharp rise in acts of GBV perpetrated against girls and women in Africa (49). In East Africa, for instance, there was a 48% increase in acts of GBV reported to the police (49). In South Africa, during the first week of the national lockdown, the South African Police Services received 2,320 complaints of GBV (49). Finally, in West Africa (Liberia), there was a 50% increase in acts of GBV during the first half of the year (49).

In all, this mini review offers valuable insights into the SRH implications of the COVID-19 pandemic for adolescents in Africa. Considering the findings of this review, corroborated by the literature, in the next section of the paper we provide recommendations and strategies to potentially reduce the impact of the pandemic adolescent SRH.

### Recommendations to Reduce the Impact of the COVID-19 Pandemic on Adolescent SRH

The increasing rates of adolescent pregnancies, together with the various barriers to accessing SRH services during the pandemic calls for “deliberate measures” (50) to address the needs of adolescents. As pointed out by Linberg, Bell and Kantor (51), “the impacts of the pandemic are still unfolding, [and] there are potential longer-term consequences that will shape [adolescent] sexual and reproductive health”.

A particular issue is the inadequate access to SRH services that adolescents, and women more generally, have including menstrual health necessities. Researchers have argued that the COVID-19 pandemic has seen a number of African countries deprioritise SRH services and relocate resources and funding to make room for Covid-19 treatment rooms (15, 52). The decision to divert critical attention from SRH has largely resulted in limited access to healthcare services for adolescents, as shown in the articles included in our sample. Similarly, to Govender and colleagues (2020), we recommend that policy makers and government adopt a human rights approach to improve accessibility to SRH services (15). We believe, as SRH is approached as a human rights issue, it will emphasize the importance of SRH and significantly improve the response of policy makers and government and access to related services during the COVID-19 pandemic (15).

In relation to teenage pregnancies, interventions are needed to ensure that adolescent mothers have access to safe and clean delivery, and antenatal and postnatal care (53). Information and education on safe and legal abortion avenues are also necessary to support young people, and their families, to make informed decisions related to their SRH (53). Given the high rates of sexual violence experienced by adolescents, concerted efforts are required to protect young girls and identify adolescents who are at increased risk for sexual and domestic violence (15). While the establishment of policies and laws to protect adolescents from harm are certainly imperative, it is not enough to ensure the health and safety of adolescents. Innovative solutions like those proposed by the United Nations Women, that prioritize the use of online or mobile technologies and “scouting activities” to identify at risk young people will further support well-established policies (15). However, as pointed out by Govender et al. (15), these innovative approaches need to be contextually sensitive, recognizing the diverse needs of adolescents in different communities.

An important space through which SRH information, along with other support details, can also be shared is through health facilities (15). The closure of these facilities during the lockdown compromised the SRH health of adolescents as they were not able to obtain contraceptives and had limited access to nurses who could provide information around protected sex, or contact details of spaces of support (15). Lindberg and colleagues further point out that pandemic-driven approaches which limited in-person contact with health staff also create barriers to seeking support (51). Lindeberg et al. (51) thus calls for a comprehensive model which makes confidential support available through multiple avenues including online, telephonically and in-person, recognizing that adolescents in rural communities might require different responses than those in more urban settings.

Further, the school has been identified as an important space through which adolescents can receive support, and through which at risk adolescents can be identified. However, we recognize that lockdown school closures create limited opportunities to provide targeted interventions to adolescents during the pandemic. In different ways school closures have contributed to the increases in adolescent pregnancy, which could subsequently also increase school dropout amongst adolescent girls (54). In this regard, the “COVID-19 pandemic could worsen existing inequalities”, as was observed during the Ebola outbreak (54). Therefore, the Empowerment and Livelihoods for Adolescents (ELA) programme in Sierra Leone amidst the Ebola outbreak focused on adolescent girls, ages 12–25 to meet outside of school to receive life skills training (55). In addition, Ni Nyampinga is a multi-platform youth brand in Rwanda that aimed to teach girls about SRH, sexual violence during COVID-19 pandemic (56). School based efforts are thus also necessary to ensure that pregnant teenagers can stay in school and return to school post-delivery to prevent further marginalization of this group.

## Conclusion

This mini review attempted to synthesize the published literature pertaining to the impact of the COVID-19 pandemic on adolescent sexual and reproductive health in Africa. To date, very few studies have specifically focused on adolescents' experiences during the pandemic, which warrants further empirical investigation. While some of the studies included in this review utilized empirical data, studies that explore the lived experiences, through qualitative work, are limited. Further research is also necessary to understand the needs of adolescents and identify avenues for targeted intervention. Furthermore, this paper is not without limitations. The paper focused on papers published within a specified timeline and within specific databases, which may have excluded relevant articles. Our inclusion criteria were also quite specific and thus papers that did not directly focus on adolescents were not included. We therefore recognize that lessons can be gleaned from the broader literature on women's sexual and reproductive health. Still, the articles included in our paper offered valuable insights into the impact of the COVID-19 pandemic on adolescent sexual and reproductive health in African countries.

## Author Contributions

CG has contributed to the conception, analysis, interpretation of data, and write up of the work. NI have contributed to the analysis, write up, collating and editing references, and revising of the work. DI has contributed to the analysis, write up, and revising of the work. All authors listed have made a substantial, direct, and intellectual contribution to the work and approved it for publication.

## Author Disclaimer

Opinions expressed and conclusions arrived at, are those of the authors and are not necessarily to be attributed to the CoE in Human Development.

## Conflict of Interest

The authors declare that the research was conducted in the absence of any commercial or financial relationships that could be construed as a potential conflict of interest.

## Publisher's Note

All claims expressed in this article are solely those of the authors and do not necessarily represent those of their affiliated organizations, or those of the publisher, the editors and the reviewers. Any product that may be evaluated in this article, or claim that may be made by its manufacturer, is not guaranteed or endorsed by the publisher.
