# Supplementary material for: Adolescent Sexual and Reproductive Health During the COVID-19 Pandemic: A Mini Review
Source: Front Reprod Health. 2022 Mar 30;4:794477. doi: 10.3389/frph.2022.794477 (PMC9580774; doi:10.3389/frph.2022.794477)
Supplement: Supplementary file 1 [file Data_Sheet_1.pdf]

Appendix A

1. Sserwanja Q, Kawuki J, Kim JH. Increased child abuse in Uganda amidst COVID-19 pandemic. Journal of Paediatrics and Child Health (2021) 57: 188–191.
2. Cancedda A, Hurnik EJ, Minardi OG, Wolsey EJ, Abdella EA. Mitigating the socio-economic impacts of COVID-19 in Ethiopia, with a focus on vulnerable groups (2020). https://www.unicef.org/ethiopia/media/3546/file/Annex%20I%20%E2%80%93%20Vulnerability%20Impact%20Fiches.pdf
3. Ndhlovu A, Thembo A. Gendered Socio-economic Implications of the COVID-19 Pandemic in Rural Zimbabwe. BizEcons Quarterly (2020) 12: 21–40
4. Eboi AA, Ismail A, Hamid NA. New Media Strategies' Model for Sexual Reproductive Health & Rights Campaigns among Young People in Informal Settlements: Mitigating the Challenges. European Journal of Molecular & Clinical Medicine (2020) 7(6): 1455-1473.
5. Addae EA. Covid-19 pandemic and adolescent health and well-being in sub-Saharan Africa: who cares? Internal Journal of Health Planning Management (2020) 36(1): 1-4. doi: 10.1002/hpm.3059
6. Ndolo P, Kivuti L, Mwai K, Chiluba B, Lukama R, Campbell E, Bloomer F. Legislative and Policy Analysis, Practice Guidelines and Related Publications within the Kenyan Context (2021). https://pure.ulster.ac.uk/en/persons/fiona-bloomer/publications/
7. Rohwerder, B. Social impacts and responses related to COVID-19 in low- and middle-income countries (2020). Institute of Development Studies, helpdesk@k4d.info [Accessed October 12, 2021].
8. Kiptoo-Tarus P. Teenage pregnancy: A psychosocial burden on girlchild education in Kenya. Research Journal in Advanced Humanities (2020) 1(2): 64-76.
9. Willie MM. Teenage Pregnancy During a Pandemic. International Journal of Women's Health Care (2020) 6(3): 218-219.
10. Rafaeli, T., & Hutchinson, G. (2020). The secondary impacts of COVID-19 on Women and Girls in Sub-Saharan Africa. Education Development Trust, helpdesk@k4d.info
11. Commonwealth Foundation. The urgent need to address the gendered impact of covid-19 on violence against women and girls in West African commonwealth countries (2021) International Federation of Women Lawyers (FIDA) Nigeria (2021). https://fida.org.ng/wp-content/uploads/2021/08/FIDA-Nigeria-Policy-Brief.pdf
12. Karp C, Moreau C, Sheehy G, Anjur-Dietrich S, Mbushi F, Muluve E, Mwanga D, Nzioki M, Pinchoff J, Austrian K. Youth Relationships in the Era of COVID-19: A Mixed-Methods Study Among Adolescent Girls and Young Women in Kenya. Journal of Adolescent Health (2021): 1-8
13. Bolarinwa OA. Factors associated with limited access to condoms and sources of condoms during the COVID-19 pandemic in South Africa (2020). Medrxiv, https://www.medrxiv.org/content/10.1101/2020.09.11.20192849v3.full
14. Sitalire, K. (2021) Assessing barriers of contraceptive uptake among adolescent girls in a rural district of Malawi [Master's thesis]. Harvard Medical School.
15. Chiweshe MK, Mushayavanhu D, Bhatasara S. An Assessment of the Formal Justice System in responding to Sexual and Gender Based Violence (SGBV), Harmful Practices (HP) and Sexual Reproductive Health Rights(SRHR) in Zimbabwe (2021). https://www.wlsazim.co.zw › access-to-justice › asses...
16. Govender K, Cowden RG, Nyamaruze P, Armstrong RM, Hatane L. Beyond the Disease: Contextualized Implications of the COVID-19 Pandemic for Children and Young People Living in Eastern and Southern Africa. Frontiers in Public Health (2020) 504(8):1-9.
17. Ameena G, Bekker LG, Van de Perre P, El-Sadr W, Ahmed K, Malahleha M, Rmaraj T, Ramokolo V, Magasana V, Gray G. Centring adolescent girls and young women in the HIV and COVID-19 responses. The Lancet (2020) 396(10266):1864-1866. doi: https://doi.org/10.1016/S0140-6736(20)32552-6
18. Allen, F. Covid-19 and Sexual and Reproductive Health of Women and Girls in Nigeria. Cosmopolitan Civil Societies: An interdisciplinary Journal (2021) 13(2): 1-11.
19. Ahinkorah BO, Hagan JE, Ameyaw EK, Seidu A-A, Schack, T. COVID-19 Pandemic Worsening Gender Inequalities for Women and Girls in Sub-Saharan Africa. Front. Glob. Womens Health (2021) 2:686984. 1-6. doi: 10.3389/fgwh.2021.68698

20. Asante KO, Quarshie EN, Andoh-Arthur J. COVID-19 school closure and adolescent mental health in sub-Saharan Africa. International Journal of Social Psychiatry (2020). 1–3
21. Govender D, Naidoo S, Taylor M. (2020). Don't let sexual and reproductive health become collateral damage in the face of the COVID-19 pandemic: A public health perspective. African Journal of Reproductive Health (2020) 24(2):56-63.
22. Adelekan T, Mihretu B, Mapanga W, Nqeketo S, Chauke L, Dwane Z, Baldwin-Ragaven L. Early Effects of the COVID-19 Pandemic on Family Planning Utilisation and Termination of Pregnancy Services in Gauteng, South Africa: March–April 2020. Wits Journal of Clinical Medicine (2020) 2(2): 145–152.
23. Shikuku D, Nyaoke I, Gichuru S, Maina O, Eyinda M, Godia P, Nyaga, L, Ameh C. Early indirect impact of COVID-19 pandemic on utilization and outcomes of reproductive, maternal, newborn, child and adolescent health services in Kenya (2020) https://www.medrxiv.org/content/10.1101/2020.09.09.20191247v1
24. Musa SS, Odey GO, Musa MK, Alhaj SM, Sunday BA, Muhammad SM, Lucero- Prisno DE. Early marriage and teenage pregnancy: The unspoken consequences of COVID-19 pandemic in Nigeria Public Health in Practice 2 (2021) 100152.
25. Masago MO, Chaka B, Alice KJS, Sheila J, Reuben, G. (2020). Effects of Covid-19 pandemic on the welfare of the Maasai girl child in Narok County, Kenya. European Journal of Social Sciences (2020) 3(3):123-141
26. Kasirye I. Expanding fiscal space for social protection: The case for adolescent-oriented services. Policy Brief (2021) 137.
27. Abuya T, Austrian K, Isaacs A, Kongwana B, Mbushi F. Experiences among adults and adolescents during the COVID-19 pandemic from four locations across Kenya—Study description and adolescents during the COVID-19 pandemic from four locations across Kenya—Study description (2020). Nairobi: Population Council.
28. Mambo SB, Sikakulya FK, Ssebuufu R, Mulago YM, Wasswa H, Kongwana B, Thompson K, Rusatira JC, Bhondoekhn F, Kamyuka LK, Akib S.O, Kirimuhuzya C, Nakawesi J, Kyamanywa P. Factors that influenced access and utilisation of sexual and reproductive health services among Ugandan youths during the COVID-19 pandemic lockdown: An online cross-sectional survey. Research Square (2020). doi: https://doi.org/10.21203/rs.3.rs-48529/v4
29. Rafaeli, T. Girl-focused life skills interventions at a distance (2020). K4D, Helpdesk Report, helpdesk@k4d.info
30. Karijo E, Wamugi S, Lemanyishoe S, Njuki J, Boit F, Kibui V, Karanja S, Abuya T. Knowledge, attitudes, practices, and the effects of COVID-19 among the youth in Kenya. BMC Public Health (2021) 21(1):1-13.
31. Eghtessadi R, Mukandavire Z, Mutenherwa F, Cuadros D, Musuka G. Safeguarding gains in the sexual and reproductive health and AIDS response amidst COVID-19: The role of African civil society. International Journal of Infectious Diseases (2020) 100:286-291.
32. Ahmed T, Rahman AE, Amole TG, Galadanci H, Matjila M, Soma-Pillay P, Gillespie BM, Arifee SE, Anumba DOC. The effect of COVID-19 on maternal newborn and child health (MNCH) services in Bangladesh, Nigeria and South Africa: call for a contextualised pandemic response in LMICs International Journal for Equity in Health (2021) 20(77): 1-6
